# ENGAGED RESEARCH: HUMILITY, LISTENING, BETTER QUESTIONS, BETTER ANSWERS

**DOI:** 10.1093/geroni/igac059.1462

**Published:** 2022-12-20

**Authors:** Andrea Gilmore-Bykovskyi

**Affiliations:** University of Wisconsin-Madison, Madison, Wisconsin, United States

## Abstract

The expansive field of aging science comprises diverse disciplines, perspectives, and approaches to address questions that extend from strengthening our understanding of biological aging to promoting wellbeing among diverse aging populations. Engaged research practices represent a cross-cutting series of diverse epistemological beliefs and pragmatic practices in the conduct of research with broad relevance to areas of inquiry across the translational aging research continuum. This presentation will describe experiences with the establishment, maintenance, and continual evolution of engaged research practices in an interdisciplinary program of health services research focused on Alzheimer’s disease and related dementias (AD/ADRD). Specifically, this presentation will discuss: a) conceptualizations of engagement and its relationship to equitable research, b) engagement as praxis for the conduct of equitable research, c) personal reflections on experiences with and the practice of humility, listening, and valuing stakeholders’ distinctive expertise, and d) practical recommendations for early career scholars approaching engaged research.

